# Is obesity a risk factor for melanoma?

**DOI:** 10.1186/s12885-023-10560-8

**Published:** 2023-02-22

**Authors:** Yuval Arbel, Yifat Arbel, Amichai Kerner, Miryam Kerner

**Affiliations:** 1 Sir Harry Solomon School of Economics and Management, Western Galilee College, Derech Hamichlalot, 2412101 Acre, Israel; 2 Department of Mathematics, Bar Ilan university, 1 Max and Anna Web Street, 5290002 Ramat Gan, Israel; 3 School of Real Estate, Netanya Academic College, 1 University Street, 4223587 Netanya, Israel; 4 The Ruth and Bruce Rapoport Faculty of Medicine, Technion – Israel Institute of Technology, 1 Efron Street, 3525422 Haifa, Israel; 5 Department of Dermatology, Emek Medical Center, 21 Yitshak Rabin Boulevard, 1834111 Afula, Israel

**Keywords:** Melanoma, Obesity, Risk factor

## Abstract

**Objectives:**

Are twofold: 1) to estimate the relationship between obesity (BMI ≥30) and the prevalence of melanoma in different US states and 2) to examine the possibility of defining a new risk group. This might enhance the possibility of detection, which in turn, might increase the survival rates of patients.

**Study design:**

A cohort Study, based on data at the US statewide level in 2011–2017, where the dependent variable (the annual new melanoma cases per 100,000 persons) is adjusted for age.

**Method:**

Quadratic regression analysis. This model permits a non-monotonic variation of obesity with new melanoma cases adjusted for age, where the control variable is the level of UV radiation.

**Results:**

Demonstrate a negative correlation between obesity and incidence of melanoma. This outcome is further corroborated for Caucasians.

**Conclusions:**

We should continue to establish primary prevention of melanoma by raising photo protection awareness and secondary prevention by promoting skin screening (by physician or self) among the entire population group in all BMI ranges. Advanced secondary melanoma prevention including noninvasive diagnosis strategies including total body photography, confocal microscopy, AI strategies should focus the high-risk sub group of Caucasians with BMI < 30.

**Supplementary Information:**

The online version contains supplementary material available at 10.1186/s12885-023-10560-8.

## Introduction

Melanoma is a skin cancer with approximately 200,000 new cases discovered annually worldwide. The greatest incidence of melanoma occurs among Australasian, North American and European, elderly and male populations. The substantial disparities in melanoma cases worldwide highlight the need for focused, aggressive prevention efforts [15].

Obesity, defined as $BMI=\frac{kg}{meter^2}\ge 30$ ([28]: Obesity, available at: https://www.who.int/health-topics/obesity#tab=tab_1 [last accessed on November 25, 2022]), is yet another risk factor for a long series of health problems, including different types of cancer [18, 23]. Yet, to date, the relationship between obesity and melanoma remains unclear. Sergentanis et al. [25] found positive association between obesity and melanoma risk among males. Lahmann et al. [16] concludes that after adjusting for sun exposure, tall stature may be a risk factor for the most common types of skin cancer BCC, SCC, and melanoma, while body mass and surface area appear irrelevant. Dusingize et al. [7] found no association between genetically predicted *BMI* and melanoma and positive association between height and melanoma.

The objective of the current study is twofold: 1) to estimate the relationship between the prevalence of melanoma adjusted for age and obesity prevalence in percentage points ($=100\times \frac{individuals\ whose\ BMI\ge 30\ in\ the\ state}{Total\ population\ in\ the\ state}$) in different US states and across time (2011–2019). 2) to examine the possibility of defining a new risk group.

The contribution of the current study lies in investigating an unexplored research question, the association between melanoma and obesity prevalence and a potential obesity survival paradox in the context of identification of new melanoma cases. Previous studies found an obesity survival paradox only in the treatment level of metastatic melanoma, but not in the identification level of skin cancer (e.g., [17, 18, 21]).^1^ The potential presence of an obesity survival paradox in the identification level of new cases of melanoma, suggesting a negative correlation between prevalence of obesity and new melanoma cases when age and *UV* radiation levels are controlled, might provide additional supporting evidence of the impact of energy balance on anti-tumor immune response through molecular, immunologic and metabolic mechanisms.

The remainder of this study is organized as follows. Section 2 gives the empirical model, the descriptive statistics of the variables incorporated in the empirical model, and the results. Section 3 reports the outcomes of the robustness test, while, sections 4 and 5 provide the discussion and summary and conclusions.

## Method

### The empirical model

Consider the following model applied to the US states:

1 $$Melanoma\ Prevalence={\alpha}_1+{\alpha}_2 UV+{\alpha}_3 Obesity\ Prevalence+ D\delta +{\mu}_1$$

Where *Melanoma Prevalence* is the annual new melanoma cases per 100,000 persons in each state adjusted for age, *UV* is the *UV* wavelet; *Obesity Prevalence* is the prevalence of obesity defined as $100\times \frac{individuals\ whose\ BMI\ge 30\ in\ the\ state}{Total\ population\ in\ the\ state}$^2^; *α*_1_, *α*_2_, *α*_3_, ⋯, *α*_9_ are parameters; *D* is a matrix of individual effect dummies (one dummy for each state, and up to 49 states); *δ* is the corresponding column vector of coefficients; *μ*_1_ is the random disturbance term, which specifies all the classical assumptions.

The empirical model contains two types of variables: 1) time varying covariates (*TVC*), which change over time (*UV*, *Obesity_Prevalence*) and 2) generic features that remains constant over time (the US state represented by the matrix *D*). One concern that should be addressed is the correlation between the *TVC* and *D*. This leads to biased and inconsistent estimates. According to Johnston and Dinardo [14], the simple way to correct this problem is the fixed-effect methodology, namely, expressing eq. (1) in terms of deviation from the mean $\overline{\left(\bullet \right)}$:

2 $$\left( Melanoma\_ Prevalence-\overline{Melanoma\_ Prevalence}\right)={\alpha}_1+{\alpha}_2\left( UV-\overline{UV}\right)+{\alpha}_3\left( Obesity\_ Prevalence-\overline{Obesity\_ Prevalence}\right)+\left(D-\overline{D}\right)\delta +\left({\mu}_1-{\overline{\mu}}_1\right)$$

It should further be noted that $\left(D-\overline{D}\right)=0$ so that the empirical model may be written as:

3 $$Melanoma\_{Prevalence}^{\ast }={\alpha}_1+{\alpha}_2{UV}^{\ast }+{\alpha}_3 Obesity\_{Prevalence}^{\ast }+{\mu}_1^{\ast }$$

Where

$$Melanoma\_{Prevalence}^{\ast }=\left( Melanoma\_ Prevalence-\overline{Melanoma\_ Prevalence}\right);\ {UV}^{\ast }=\left( UV-\overline{UV}\right);\ Obesity\_{Prevalence}^{\ast }=\left( Obesity\_ Prevalence-\overline{Obesity\_ Prevalence}\right)$$

To permit quadratic relationships, we also supplement the following extensions to the parameters of the empirical model:

4 $${\alpha}_1={\beta}_1$$

5 $${\alpha}_2={\beta}_2+{\beta}_3{UV}^{\ast }+{\beta}_4 Obesity\_{Prevalence}^{\ast }+{\beta}_5{\left( Obesity\_{Prevalence}^{\ast}\right)}^2+{\beta}_6 UV\times {\left( Obesity\_{Prevalence}^{\ast}\right)}^2$$

6 $${\alpha}_3={\beta}_7+{\beta}_8 Obesity\_{Prevalence}^{\ast }+{\beta}_9{\left({UV}^{\ast}\right)}^2$$

Where *β*_1_, *β*_2_, *β*_3_, ⋯, *β*_9_ are parameters. Substitution of (4)–(6) in (3) yields:

7 $$Melanoma\_{Prevalence}^{\ast }={\beta}_1+{\beta}_2{UV}^{\ast }+{\beta}_3{\left({UV}^{\ast}\right)}^2+{\beta}_4{UV}^{\ast}\times Obesity\_{Prevalence}^{\ast }+{\beta}_5{UV}^{\ast}\times {\left( Obesity\_{Prevalence}^{\ast}\right)}^2+{\beta}_6{\left({UV}^{\ast}\right)}^2\times {\left( Obesity\_{Prevalence}^{\ast}\right)}^2+{\beta}_7 Obesity\_{Prevalence}^{\ast }+{\beta}_8{\left( Obesity\_{Prevalence}^{\ast}\right)}^2+{\beta}_9 Obesity\_{Prevalence}^{\ast}\times {\left({UV}^{\ast}\right)}^2+{\mu}_1^{\ast }$$

### Descriptive statistics

Table 1 reports the descriptive statistics of variables that were incorporated in the empirical model. The average number of annual new melanoma cases adjusted for age is 20.74–22.62 cases and the standard deviation is 4.41–5.23 per 100,000 persons (*Melanoma PrevalenceMelanoma Prevalence*). The null hypothesis of zero new annual melanoma cases per 100,000 persons is clearly rejected for both periods (2005–2010 and 2010–2015). For 2011–2015, the 99% confidence interval is [21.75, 23.48] and for 2005–2010 the 99% confidence interval is [20.05, 21.42]. A possible implication of these figures is a growth in the average number of new melanoma cases over time. The minimum number of annual new melanoma cases is 7.60 and the maximum is 42.90.

**Table 1:** Descriptive statistics

Pooled Sample (2005–2015)						
**Variable**	**Description**	**Obs.**	**Mean**	**Std.**	**Min**	**Max**
Melanoma Prevalence	Annual new melanoma cases per 100,000 persons adjusted for age	527	21.61	4.90	7.60	42.70
Obesity Prevalence	Prevalence of obesity in US states multiplied by 100	527	27.74	3.92	16.90	40.80
*UV*	Ultraviolet wavelet measured in nanometers (nm)	527	124.15	27.22	83.00	186.00
2011–2015						
**Variable**	**Description**	**Obs.**	**Mean**	**Std.**	**Min**	**Max**
Melanoma Prevalence	Annual new melanoma cases per 100,000 persons adjusted for age	245	22.62	5.23	7.60	42.70
Obesity Prevalence	Prevalence of obesity in US states multiplied by 100	245	28.67	3.43	20.20	36.20
*UV*	Ultraviolet wavelet measured in nanometers (nm)	245	125.89	27.59	85.00	186.00
2005–2010						
**Variable**	**Description**	**Obs.**	**Mean**	**Std.**	**Min**	**Max**
Melanoma Prevalence	Annual new melanoma cases per 100,000 persons adjusted for age	282	20.74	4.41	7.80	34.10
Obesity Prevalence	Prevalence of obesity in US states multiplied by 100	282	26.93	4.13	16.90	40.80
*UV*	Ultraviolet wavelet measured in nanometers (nm)	282	122.64	26.85	83.00	185.00

The table consists of 40–49 US states between 2005 and 2015. As of 2011 the definition of obesity was changed and, in contrast to 2005–2010, the prevalence of obesity increased over time in 49 US states

Referring to the prevalence of obesity in US states, namely, percent of the population whose body mass index (*BMI*= $\frac{kg}{meter^2}$) is higher than 30, the average prevalence is 26.93–28.67% and the standard deviation is 3.43–4.13% (*Obesity Prevalence*). The null hypothesis of zero prevalence of obesity is clearly rejected for both periods. For 2011–2015, the 99% confidence interval is [28.10, 29.24] and for 2005–2010 the 99% confidence interval is [26.29, 27.57]. Again, a potential implication is a growth in obesity prevalence over time. The minimum number of annual new obesity cases is 16.90 and the maximum is 40.80.

Finally, referring to ultraviolet (*UV*) radiation in US states, measured in nanometers (nm), where the shorter the wavelet the higher the level of *UV* radiation, the average *UV* radiation is 122.64–125.89 nm. and the standard deviation is 26.85–27.59. Furthermore, the minimum *UV* radiation is 83 and the maximum is 186.

Table 2 reports the pairwise Pearson correlation matrix. As anticipated, the table shows negative correlations between higher wavelet of *UV* radiation and new melanoma cases and positive correlation between *UV* radiation and prevalence of obesity. For all the correlations, the null hypothesis of zero correlation is rejected at the 10–1% levels.

**Table 2:** Pearson correlation matrix

Full Sample (2005–2015 and 527 Obs × Years)			
	Melanoma Prevalence	*UV*	Obesity Prevalence
Melanoma Prevalence	1.0000		
*UV*	−0.2784***	1.0000	
	(< 0.01)		
Obesity Prevalence	−0.1274***	0.1665***	1.0000
	(0.0034)	(0.0001)	
After Modification of Obesity Definition (2011–2015 and 245 Obs × Years)			
	Melanoma Prevalence	*UV*	Obesity Prevalence
Melanoma Prevalence	1.0000		
*UV*	−0.3048***	1.0000	
	(< 0.01)		
Obesity Prevalence	−0.1246*	0.1520**	1.0000
	(0.0514)	(0.0172)	
Before Modification of Obesity Definition (2005–2010 and 282 Obs × Years)			
	Melanoma Prevalence	*UV*	Obesity Prevalence
Melanoma Prevalence *UV*	1.0000		
	−0.2879***	1.0000	
	(< 0.01)		
Obesity Prevalence	−0.2280***	0.1630***	1.0000
	(0.0001)	(0.0061)	

*P*-values for the rejection of zero Pearson correlations are given in parentheses. *p < 0.1; **p < 0.05; ***p < 0.01

## Results

The first step of the analysis would be to demonstrate that *Melanoma Prevalence*, *UV*, *Obesity Prevalence* are time varying covariates, namely they change over time, otherwise it is impossible to use the fixed-effect methodology due to perfect collinearity. Table 3 reports the outcomes and demonstrate that the projected new melanoma cases (adjusted for age) in 2011 is 21 cases per 100,000 persons. During 2011–2015, the prevalence of new melanoma cases is expected to *rise* by 0.664 per annum (*p* = 0.00436), so that in 2015 this projected prevalence becomes 24 cases per 100,000 persons. The expected prevalence of obesity (*BMI* ≥ 30) – after the change of definition – is 27.73% in 2011. During 2011–2015, and in contrast to 2005–2010, the prevalence of obesity is expected to *rise* by 0.468 per annum (*p* = 0.00211), so that in 2015 this projected prevalence becomes 29.61%. The *UV* wavelet remains unchanged over time (*p* = 0.921) with a slight tendency to drop.

**Table 3:** Time varying covariates

2005–2015			
Variables	Melanoma Prevalence	*UV*	Obesity Prevalence
Constant	19.77***	121.3***	26.23***
	(< 0.01)	(< 0.01)	(< 0.01)
(Year-2005)	0.363***	0.555	0.297***
	(5.06 × 10^−8^)	(0.139)	(2.32 × 10^−8^)
Observations	527	527	527
F(1,525)	30.58***	2.20	32.18***
2011–2015			
Variables	Melanoma Prevalence	*UV*	Obesity Prevalence
Constant	21.29***	126.1***	27.73***
	(< 0.01)	(< 0.01)	(< 0.01)
(Year-2011)	0.664***	−0.124	0.468***
	(0.00436)	(0.921)	(0.00211)
Observations	245	245	245
Years	5	5	5
F(1,243)	8.13***	0.01	9.45***
2005–2010			
Variables	Melanoma Prevalence	*UV*	Obesity Prevalence
Constant	20.03***	120.7***	26.67***
	(< 0.01)	(< 0.01)	(< 0.01)
(Year-2005)	0.281*	0.769	0.104
	(0.0634)	(0.405)	(0.463)
Observations	282	282	282
Years	6	6	6
F(1,280)	3.47*	0.70	0.54

*P*-values are given in parentheses. *p < 0.1; **p < 0.05; ***p < 0.01

Table 4 reports the outcomes of the regression analyses obtained from eq. (7). Given the methodological changes in obesity measurement since 2011 (e.g., https://www.cdc.gov/obesity/data/prevalence-maps.html [last accessed on December 22, 2022]: “†Prevalence estimates reflect BRFSS methodological changes started in 2011. These estimates should not be compared to prevalence estimates before 2011”), the sample is divided to two parts (2005–2010 and 2011–2015).

**Table 4:** Regression analysis: annual new melanoma cases per 100,000 persons

Years		2005–2015	2011–2015	2005–2010
Variables	Coef.	Melanoma Prevalence	Melanoma Prevalence	Melanoma Prevalence
Constant	*β*_1_	537.4**	− 303.4***	171.5***
		(0.0281)	(0.00338)	(0.000255)
*UV*	*β*_2_	−8.798**	2.005***	−2.254***
		(0.0237)	(0.00513)	(0.00253)
*UV*^2^	*β*_3_	0.0348**	–	0.00857***
		(0.0190)	–	(0.00287)
*UV*× Obesity_Prevalence	*β*_4_	0.519*	−0.142***	0.0857***
		(0.0635)	(0.00544)	(0.00217)
*UV*×(Obesity_Prevalence)^2^	*β*_5_	−0.00710	0.00244***	–
		(0.155)	(0.00728)	–
*UV*^2^ × (Obesity_Prevalence)^2^	*β*_6_	2.87 × 10 ^−5^	–	–
		(0.131)	–	–
Obesity_Prevalence	*β*_7_	−29.58*	23.34***	−5.634***
		(0.0934)	(0.00167)	(0.00133)
(Obesity_Prevalence)^2^	*β*_8_	0.391	−0.405***	–
		(0.216)	(0.00215)	–
Obesity_Prevalence × *UV*^2^	*β*_9_	−0.00207*	–	−0.000332***
		(0.0516)	–	(0.00205)
Method		Fixed-Effect	Fixed-Effect	Fixed-Effect
Corr(*X*,*U*)		−0.2288	−0.1047	−0.4116
Observations		527	245	282
Years		2005–2015	2011–2015	2005–2010
Number of Years		11	5	6
Number of States		40–49	49	40–49
R-Squared		0.172	0.172	0.168

The table provides the estimation outcomes obtained from eq. (7). Given the change in obesity measurement reported by the CDC, we separated the analysis to two segments (2005–2010 and 2011–2015). While the left column gives the outcomes of the pooled sample (2005–2015), the right [middle] column displays the outcomes of the first [second] segment, namely 2005–2010 [2011–2015]. *P*-values are given in parentheses. *p < 0.1; **p < 0.05; ***p < 0.01

**Fig. 1:**
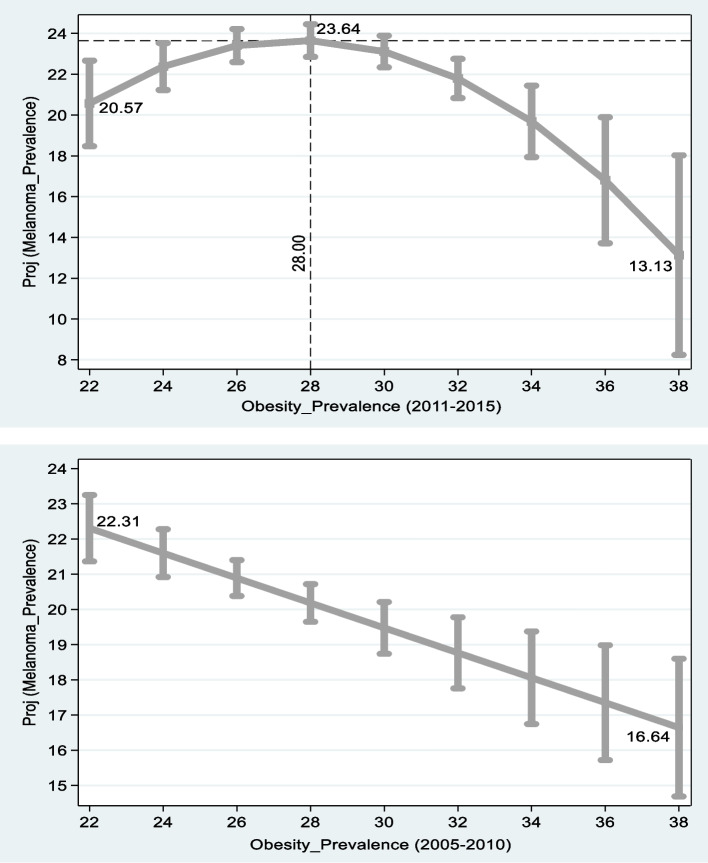
Projected Rates of New Melanoma Cases vs. Prevalence of Obesity in US States. Notes: Sources: 1) Center for Disease Control and Prevention (CDC) [5]: Overweight & Obesity, Available at: https://www.cdc.gov/obesity/data/prevalence-maps.html 2) Center for Disease Control and Prevention (CDC) [6]: United States Cancer Statistics, available at: https://www.cdc.gov/cancer/uscs/ 3) [19], available at: https://www.cpc.ncep.noaa.gov/. Melanoma Prevalence = annual new melanoma cases per 100,000 persons adjusted for age. The graphs are based on the middle [right] columns of Table 4. The difference between the lower and upper graph emanates from the methodological changes in obesity measurement by the CDC starting from 2011

**Fig. 2:**
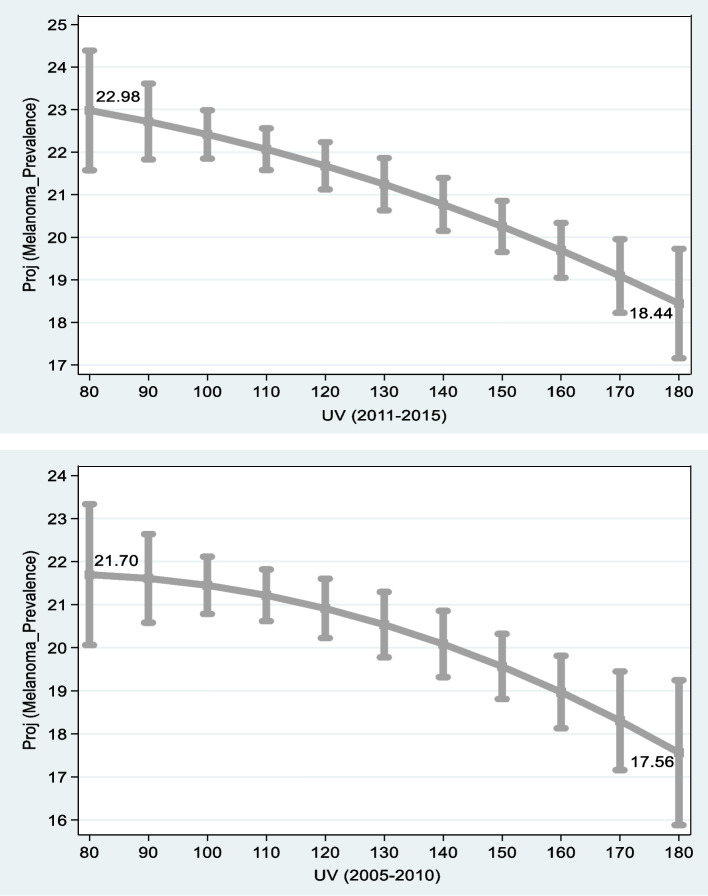
Projected Rates of New Melanoma Cases vs. UV wavelet in US States. Notes: Sources: 1) Center for Disease Control and Prevention (CDC) [5]: Overweight & Obesity, Available at: https://www.cdc.gov/obesity/data/prevalence-maps.html 2) Center for Disease Control and Prevention (CDC) [6]: United States Cancer Statistics, available at: https://www.cdc.gov/cancer/uscs/ 3) [19], available at: https://www.cpc.ncep.noaa.gov/. Melanoma Prevalence = annual new melanoma cases per 100,000 persons adjusted for age. The shorter the wavelet the higher the level of *UV* radiation. The graphs are based on the middle and right columns of Table 4

Figures 1 and 2 are based on the right and middle columns of Table 4. The upper graph in Fig. 1 shows that for states in which the prevalence of obesity is 22–28%, projected new melanoma cases per 100,000 persons *rise* from 20.57 to 23.64. For states in which the prevalence of obesity is 28–38%, projected new melanoma cases per 100,000 persons *drop* from 23.64 to 13.13. The lower graph demonstrates a projected *drop* from 22.31 cases per 100,000 persons in states, where 22% of the population suffers from obesity to 16.64 in states where 38% of the population suffers from obesity.

As anticipated, Fig. 2 demonstrates that the shorter the wavelet, the higher the level of new melanoma cases per 100,000 persons. In *UV* wavelet of 80 nm – the anticipated level of new melanoma cases per 100,000 persons is 21.70–22.98. The projected number of cases *drops* to 17.56–18.44 new melanoma cases per 100,000 persons in *UV* wavelet of 180 nm.

## Robustness test

One concern associated with previous sections is the lack of confounders such as complexion. To address this concern, we ran a robustness test. Consider the following model applied to 46 states and to 2011–2019 (prior to the outburst of the COVID19 pandemic and after the modification of obesity measurement reported by the CDC)^3^:

8 $$Melanoma\_ Prevalence={\delta}_1\left( Year-2011\right)+{\delta}_2 Obesity\_{Prevalence}^2+{\delta}_3 White\times Obesity\_{Prevalence}^2+{\delta}_4 Obesity\_ Prevalence+{\delta}_5 White\times Obesity\_ Prevalence+{\delta}_6 White+{\delta}_7+{\epsilon}_1$$

Where *Melanoma* _ *Prevalence* is the dependent variable, (*Year* − 2011),^4^*Obesity* _ *Prevalence*^2^, *Obesity* _ *Prevalence*, *White* are the independent variables (*White* = 1 for white population and zero for black population), *δ*_1_, *δ*_2_, *δ*_3_, *δ*_4_, *δ*_5_, *δ*_6_, *δ*_7_ are parameters and *ϵ*_1_ is the random disturbance term. Results obtained from this empirical model are reported in Tables 5 and 6.

**Table 5:** Robustness test: melanoma and obesity prevalence: white vs. black population

	Full Model	Stepwise Model
VARIABLES	Melanoma Prevalence	Melanoma Prevalence
(*Year* − 2011)	0.371***	0.339***
	(1.62 × 10^−5^)	(5.54 × 10^− 5^)
*Obesity* _ *Prevalence*^2^	− 0.00284	–
	(0.640)	–
*White* × *Obesity* _ *Prevalence*^2^	−0.0168*	− 0.00638***
	(0.0689)	(2.93 × 10^−7^)
*Obesity* _ *Prevalence*	0.177	–
	(0.690)	–
*White* × *Obesity* _ *Prevalence*	0.514	–
	(0.366)	–
Constant	−2.873	−0.205
	(0.722)	(0.653)
*White*	24.71***	30.88***
	(0.00806)	(< 0.01)
Observations	843	843
R-squared	0.819	0.818

The table provides the outcomes obtained from the full model given by eq. (8) and those obtained from the stepwise procedure. The latter is based on iterations, in each of which the independent variable whose coefficient has the highest *p*-value is omitted. These iterations are continued until the final model includes only independent variables whose coefficients are lower than a pre-determined threshold *p*-value, where the conventional one is *p* < 0.05. *P*-values are given in parentheses. *p < 0.1; **p < 0.05; ***p < 0.01

**Table 6:** Regression analysis: white vs. black across time

	Full Model	Stepwise Model	Full Model	Stepwise Model
VARIABLES	Melanoma Prevalence	Melanoma Prevalence	Obesity Prevalence	Obesity Prevalence
Constant	0.964*	1.158***	36.23***	35.90***
	(0.0972)	(0.000241)	(< 0.01)	(< 0.01)
*White*	24.34***	24.14***	−10.23***	−9.617***
	(< 0.01)	(< 0.01)	(< 0.01)	(< 0.01)
(*Year* − 2011)	0.0481	–	0.382***	0.465***
	(0.692)	–	(7.05 × 10^−5^)	(< 0.01)
*White* × (*Year* − 2011)	0.336**	0.384***	0.152	–
	(0.0418)	(0.000584)	(0.242)	–
Observations	843	843	843	843
R-squared	0.813	0.813	0.512	0.511

The melanoma prevalence variable is adjusted for age. P-values are given in parentheses. *p < 0.1; **p < 0.05; ***p < 0.01

The table provides the outcomes obtained from the full model given by eq. (8) and those obtained from the stepwise procedure. The latter is based on iterations, in each of which the independent variable whose coefficient has the highest *p*-value is omitted. These iterations are continued until the final model includes only independent variables whose coefficients are lower than a pre-determined threshold *p*-value, where the conventional one is *p* < 0.05.

The outcomes demonstrate a very good fit of the data to this interaction model (*R*^2^ = 0.818 − 0.819). The implication is that 81.8–81.9% of the variance of the dependent variable, namely, melanoma prevalence, is explained by the independent variables at a statewide level. Further results suggest that the baseline projected melanoma prevalence at sample states with zero prevalence of obesity in 2011 is 24.71 (*p* = 0.00806) -30.88 (*p* < 0.01) new melanoma patients per 100,000 persons for both populations. The annual growth in projected melanoma prevalence is 3.39 (*p* = 5.54 × 10^− 5^) – 3.71 (*p* = 1.62 × 10^− 5^) melanoma patients per 100,000 persons in the population.

**Fig. 3:**
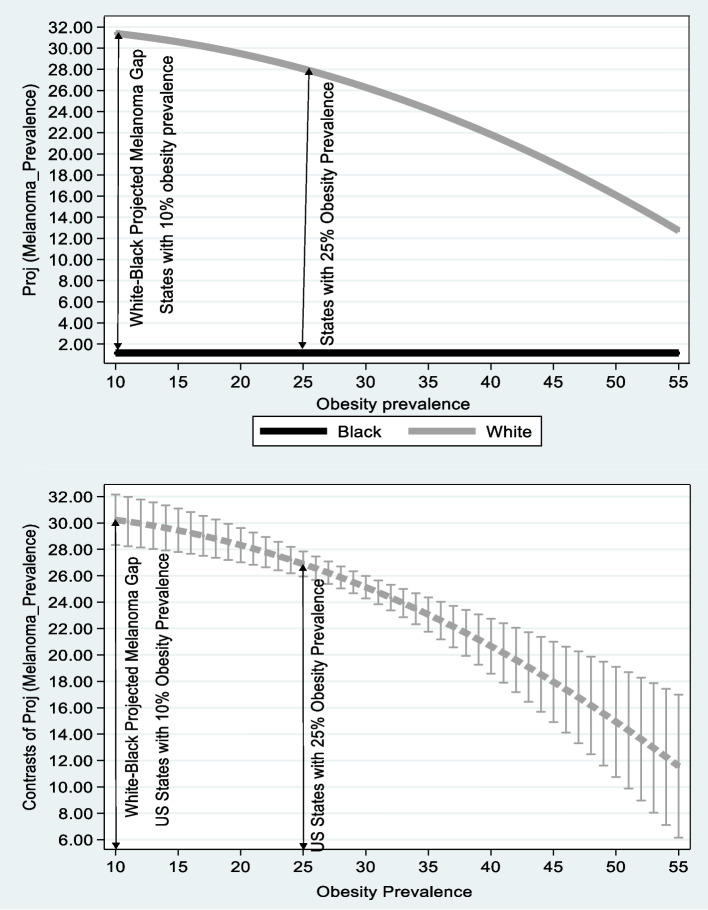
Melanoma and Obesity Prevalence: White vs. Black Population. Notes: Based on the right column of Table 5. The vertical axis at the top figure reflects the projected melanoma prevalence adjusted for age. The vertical axis at the bottom figure measures the white, black projected melanoma prevalence differences adjusted for age and their 95% confidence intervals for the same obesity prevalence. The horizontal axes in both figures measure obesity prevalence at the statewide level

Figure 3 is based on the right column of Table 5. The vertical axis at the top figure reflects the projected melanoma prevalence adjusted for age. The vertical axis at the bottom figure measures the white, lack projected melanoma prevalence differences adjusted for age and their 95% confidence intervals for the same obesity prevalence. The horizontal axes in both figures measure obesity prevalence at the statewide level.^5^

The top figure clearly indicates that on the one hand, for the white population, projected melanoma prevalence drops from 32 patients per 100,000 persons where obesity prevalence is 10% to 12 patients per 100,000 persons where obesity prevalence is 55%. On the other hand, for the black population, projected melanoma prevalence remains stable regardless of obesity prevalence at the level of below 2 patients per 100,000 black persons. Moreover, the bottom figure demonstrates that as the lower bound of the 95% confidence interval is above zero,

for each obesity prevalence, the positive white-black projected melanoma gap is preserved. Differently put, The lower figure shows that for each obesity prevalence the gap is statistically significant.

Indeed, Caucasian-African Americans dissimilarities in melanoma prevalence were found in other studies. Based on the Oklahoma Central Cancer Registry in 2000–2008, Baldwin et al. [4] suggest that white non-Hispanics in Oklahoma have the highest period prevalence (*p* < 0.0001) among the racial strata. In their review, Higgins et al. [12] mention the fact that compared to Caucasians, melanoma has unique demographic, clinical, and genetic features among African American populations.

## Discussion

Melanoma is a multi-factorial disease, which depends on environmental characteristics, such as excess exposure to UV radiation from sun or artificial tanning procedures, genetic factors, such as phenotype of Fitzpatrick 1–3 skin type, BRAF activating mutations and tumor microenvironment. Melanoma treatment is based on these multi-factors, starting from total prevention by avoidance from exposure to UV radiation of the sun, and with the progress of the disease targeted biological treatment, such as, BRAF inhibitors, MEK inhibitors, immune checkpoint strategies like CTLA4 antibodies [11] and immune checkpoint strategies like PD-1 antibodies [10, 27].

The prognosis of a melanoma patient is directly related to the diagnostic stage of the disease. Stage 4 melanomas or those thicker than 4 mm have a poor prognosis (5-year survival: 15.7 and 56.6%) [22]. In contrast, thin melanomas thinner than1 mm are associated with a very good prognosis. Prognosis varies from a disease-free survival of close to 100% to about 70% [8].

Early detection of melanoma reduces morbidity and mortality by reducing the extent of surgical removal, reducing the potential side effects of systemic therapies and reduces the care costs [20].

Definition of melanoma risk groups and prevention efforts might prove to be important and may include public specific campaigns; noninvasive skin imaging technologies; using deep learning and artificial intelligence to improve melanoma early detection

In contrast to previous studies, and based on data at the US statewide level, our findings suggest a negative correlation between prevalence of obesity and incidence of melanoma, when age and *UV* radiation levels are controlled. The implication of these findings might be an obesity paradox, namely, the counter-intuitive possibility that higher prevalence of obesity *reduces* the risk of melanoma. Indeed, evidence for an obesity survival paradox has previously been identified. For example, Stefan et al. [26] notes: “Conversely, an obesity survival paradox has been observed in patients with pneumonia. That is, despite the increased risk of pneumonia and difficulties of intubation and mask ventilation, the risk of death in patients with obesity and pneumonia might be decreased. Potentially counter-balancing effects of obesity might include the more aggressive treatment provided to these patients, their increased metabolic reserve or other unidentified factors” (page 341). Likewise, Arbel et al. [2] found evidence that both projected rates of infection and mortality from coronavirus disease *drop* with elevated prevalence of obesity in US states. In addition, Petrelli et al. [21] suggest that: “patients with obesity and lung cancer, renal cell carcinoma, and melanoma had a lower risk of death than patients with the same cancers without obesity.” (Abstract)

## Summary and conclusions

The objective of the current study is twofold: 1) to estimate the relationship between obesity (*BMI* ≥ 30) and the prevalence of melanoma in different US states and 2) to examine the possibility of defining a new risk group.

Our findings add to the current research by demonstrating, in contrast to the existing literature, a potential obesity survival paradox in identification level of new cases of melanoma. Previous studies found evidence of an obesity survival paradox only in treatment levels, but not in identification levels of skin cancer (e.g., [17, 18, 21]).

A potential explanation underlying the interaction between obesity and melanoma is the impact of energy balance on anti-tumor immune response. Another possible cause through which obesity might reduce the prospects of skin cancer is social and behavioral mechanisms, e.g., that obese persons are less exposed to the sun due to lower levels of physical activity and walk outside home. This explanation may be supported by Dusingize et al. [7], who found no association between genetically predicted *BMI* and melanoma; and by the findings that an increased risk of obesity has been reported among those with low vitamin D levels, which, in turn, may be produced from the sun [3, 30]. Another support comes from [9, 24]; and [1]. This strand of the literature deals with the spatial context of the relationship between obesity and lack of physical activity, and, in particular, car-oriented communities, which, in turn, reduce the opportunities for walking outside home.

In sum, the implication of these findings might be an obesity survival paradox, namely, the counter-intuitive possibility that a higher prevalence of obesity reduces the risk of melanoma. This outcome is further corroborated for Caucasians.

The public policy repercussions of our study are the following: we should continue to establish primary prevention of melanoma by raising photo protection awareness and secondary prevention by promoting skin screening (by physician or self) among the entire population group in all BMI ranges. Advanced secondary melanoma prevention including noninvasive diagnosis strategies including total body photography, confocal microscopy, AI strategies should focus the high-risk sub group of Caucasians with BMI < 30.

A potential limitation of our study is the employment of aggregated data at the US statewide level. Consequently, future studies should investigate this research question further by using a lower grid of data at a personal micro-level.

## Supplementary Information

**Additional file 1: Appendix S1.** Four US Ethnic Groups.

## Data Availability

The datasets generated and/or analyzed during the current study are available in the following link: https://www.cdc.gov/cancer/uscs/.

## Footnotes

1 As McQuade et al. [18] suggest: “Although the impact of obesity associated inflammation on carcinogenesis has been well studied, the impact of energy balance on anti-tumor immune response has not been examined to date and should be investigated as a potential explanation underlying the observed interaction between *BMI* and both targeted and immune therapy.” (page 320).

2 This calculation transforms the variable to percentage points, so that obesity prevalence of *X* percent in the population (0 ≤ *X* ≤ 100) equals *X*%.

3 The information was obtained from Center for Disease Control and Prevention (CDC) [6]: United States Cancer Statistics, available at: https://www.cdc.gov/cancer/uscs/ [last accessed on December 22, 2022]. The reason for the white-black choice is our findings these groups have the most extreme differences in terms of melanoma prevalence. In this context, see the graph at Additional file 1: Appendix S1.

4 Incorporation of the time variable (*Year* − 2011) addresses the possibility of spurious or nonsense correlation in time series analysis. According to Johnston and Dinardo [14], series, responding to unrelated mechanisms, such as, death rates in England and Wales and the proportion of all marriages solemnized in the Church of England from 1866 to 1911 [29], may display contemporaneous upward or downward movement. This problem may be addressed by fitting trends to such series. Regression outcomes reported in Table 6, demonstrate that while melanoma prevalence rises by 0.336−0.384% per annum only for the white population, obesity prevalence increases by 0.382−0.465% per annum only for the black population.

5 Calculations of the figures were carried out in Stata software package version 16 via the following commands: • margins White, at(Obesity_prevalence==(10(1)55)) • marginsplot, noci • margins White, at(Obesity_prevalence==(10(1)55)) contrast • marginsplot The “margins” and “marginsplot” commands come after the corresponding regression analysis.

## References

[1] Arbel Y, Fialkoff C, Kerner A, Kerner M (2020). Can reduction in infection and mortality rates from coronavirus be explained by an obesity survival paradox? An analysis at the US statewide level. Int J Obes.

[2] Arbel Y, Fialkoff C, Kerner A (2020). The chicken and egg problem: obesity and the urban monocentric model. The J Real Estate Financ Econ.

[3] Asmaa MSG, Abd El-Aziz EA (2017). Vitamin D reduces high-fat diet induced weight gain and C-reactive protein, increases interleukin-10, and reduces CD86 and caspase-3. Pathophysiology.

[4] Baldwin J, Janitz AE, Erb-Alvarez J, Snider C, Campbell JE (2016). Prevalence and mortality of melanoma in Oklahoma among racial groups, 2000-2008. J Oklahoma State Med Assoc.

[5] Center for Disease Control and Prevention (CDC) (n.d.-a): Overweight & Obesity, Available at: https://www.cdc.gov/obesity/data/prevalence-maps.html Accessed 22 Dec 2022.

[6] Center for Disease Control and Prevention (CDC) (n.d.-b): United States Cancer Statistics, Available at: https://www.cdc.gov/cancer/uscs/ Accessed 22 Dec 2022.

[7] Dusingize JC, Olsen CM, An J, Pandeya N, Law MH, Thompson BS, Goldstein AM, Iles MM, Webb PM, Neale RE, Ong J-S, MacGregor S, Whiteman DC (2020). Body mass index and height and risk of cutaneous melanoma: Mendelian randomization analyses. Int J Epidemiol.

[8] Elder DE (2011). Thin melanoma. Arch Pathol Laboratory Med.

[9] Ewing R, Meakins G, Hamidi S, Nelson AC (2014). Relationship between urban sprawl and physical activity, obesity, and morbidity – update and refinement. Health Place.

[10] Garbe C, Amaral T, Peris K, Hauschild A, Arenberger P, Basset-Seguin N, Bastholt L, Bataille V, del Marmol V, Dréno B, Fargnoli MC, Forsea A-M, Grob J-J, Hoeller C, Kaufmann R, Kelleners-Smeets N, Lallas A, Lebbé C, Lytvynenko B, Malvehy J (2022). European consensus-based interdisciplinary guideline for melanoma. Part 2: treatment - update 2022. Eur J Cancer.

[11] Grimaldi AM, Cassidy PB, Leachmann S, Ascierto PA. Novel approaches in melanoma prevention and therapy. Adv Nutr Cancer. 2014:443–55. 10.1007/978-3-642-38007-5_25. 10.1007/978-3-642-38007-5_25 24114495

[12] Higgins S, Nazemi A, Feinstein S, Chow M, Wysong A (2019). Clinical presentations of melanoma in African Americans, Hispanics, and Asians. Dermatol Surg.

[13] Ichii-Jones F, Lear JT, Heagerty AH, Smith AG, Hutchinson PE, Osborne J, Bowers B, Jones PW, Davies E, Ollier WE, Thomson W, Yengi L, Bath J, Fryer AA, Strange RC (1998). Susceptibility to melanoma: influence of skin type and polymorphism in the melanocyte stimulating hormone receptor gene. J Investig Dermatol.

[14] Johnston J, Dinardo J (1997). Econometric Methods.

[15] Karimkhani C, Green AC, Nijsten T, Weinstock MA, Dellavalle RP, Naghavi M, Fitzmaurice C (2017). The global burden of melanoma: results from the global burden of disease study 2015. Br J Dermatol.

[16] Lahmann PH, Hughes MCB, Williams GM, Green AC (2016). A prospective study of measured body size and height and risk of keratinocyte cancers and melanoma. Cancer Epidemiol.

[17] Lennon H, Sperrin M, Badrick E, Renehan AG (2016). The obesity paradox in cancer: a review. Curr Oncol Rep.

[18] McQuade JL, Daniel CR, Hess KR (2018). Association of body-mass index and outcomes in patients with metastatic melanoma treated with targeted therapy, immunotherapy, or chemotherapy: a retrospective, multicohort analysis. Lancet Oncol.

[19] National Weather Service, Climate Prediction Center (n.d.), Available at: https://www.cpc.ncep.noaa.gov/products/stratosphere/uv_index/uv_annual.shtml Accessed 22 Dec 2022.

[20] Petrie T, Samatham R, Witkowski AM, Esteva A, Leachman SA (2019). Melanoma early detection: big data, bigger picture. J Investig Dermatol.

[21] Petrelli F, Cortellini A, Indini A, Tomasello G, Ghidini M, Nigro O, Salati M, Dottorini L, Iaculli A, Varricchio A, Rampulla V, Barni S, Cabiddu M, Bossi A, Ghidini A, Zaniboni A (2021). Association of obesity with survival outcomes in patients with cancer: a systematic review and meta-analysis. JAMA Netw Open.

[22] Pollack LA, Li J, Berkowitz Z, Weir HK, Wu X-C, Ajani UA, Ekwueme DU, Li C, Pollack BP (2011). Melanoma survival in the United States, 1992 to 2005. J Am Acad Dermatol.

[23] Renehan AG, Tyson M, Egger M, Heller RF, Zwahlen M (2008). Body-mass index and incidence of cancer: a systematic review and meta-analysis of prospective observational studies. Lancet.

[24] Sallis JF, Cerin E, Conway TL, Adams MA, Frank LD, Pratt M, Salvo D (2016). Physical activity in relation to urban environments in 14 cities worldwide: a cross-sectional study. Lancet.

[25] Sergentanis TN, Antoniadis AG, Gogas HJ (2013). Obesity and risk of malignant melanoma: a meta-analysis of cohort and case-control studies. Eur J Cancer.

[26] Stefan N, Birkenfeld AL, Schulze MB (2020). Obesity and impaired metabolic health in patients with COVID-19. Nat Rev Endocrinol.

[27] Skudalski L, Waldman R, Kerr PE, Grant-Kels JM (2022). Melanoma: an update on systemic therapies. J Am Acad Dermatol.

[28] World Health Organization (n.d.): Obesity, Available at: https://www.who.int/health-topics/obesity#tab=tab_1 Accessed 25 Nov 2022.

[29] Yule GU (1926). Why do we Sometimes get Nonsense-Correlations between Time-Series?--A Study in Sampling and the Nature of Time-Series. J R Stat Soc.

[30] Zhang M, Li P, Zhu Y, Chang H, Wang X, Liu W, Zhang Y, Huang G (2015). Higher visceral fat area increases the risk of vitamin D insufficiency and deficiency in Chinese adults Nutr. Metab.

