# Supplementary material for: Is obesity a risk factor for melanoma?
Source: BMC Cancer. 2023 Feb 22;23:178. doi: 10.1186/s12885-023-10560-8 (PMC9944773; doi:10.1186/s12885-023-10560-8)
Supplement: Supplementary file 1 — Additional file 1: Appendix S1. Four US Ethnic Groups. [file 12885_2023_10560_MOESM1_ESM.docx]

**Appendix S1:** Four US Ethnic Groups

Notes: The graph is based on the outcomes obtained from the fractional probit regression
